# Dome versus single-cut osteotomies for correction of long bone deformities—technical considerations

**DOI:** 10.1038/s41598-024-62410-7

**Published:** 2024-06-04

**Authors:** Christoph Zindel, Sandro Hodel, Philipp Fürnstahl, Andreas Schweizer, Sandro F. Fucentese, Lazaros Vlachopoulos

**Affiliations:** 1 Department of Orthopedics, Balgrist University Hospital, University of Zurich, Forchstrasse 340, 8008 Zurich, Switzerland; 2 Research in Orthopedic Computer Science (ROCS), Balgrist University Hospital, University of Zurich, Balgrist CAMPUS, Zurich, Switzerland

**Keywords:** Anatomy, Medical research, Engineering

## Abstract

Corrective osteotomy allows to improve joint loading, pain and function. In complex deformities, the biggest challenge is to define the optimal surgical solution, while considering anatomical, technical and biomechanical factors. While the single-cut osteotomy (SCOT) and focal dome osteotomy (FDO) are well-established treatment options, their mathematical relationship remain largely unclear. The aim of the study was (1) to describe the close mathematical relationship between the SCOT and FDO and (2) to analyze and introduce a novel technique—the stepped FDO—as a modification of the classic FDO. The mathematical background and relationship of SCOT and FDO are described for the example of a femoral deformity correction and visualized using a 3D surface model taking into account the benefits for the clinical application. The novel modifications of the stepped FDO are introduced and its technical and clinical feasibility demonstrated. Both, SCOT and FDO, rely on the same deformity axis that defines the rotation axis *k* for a 3D deformity correction. To achieve the desired correction using a SCOT, the resulting cutting plane is perpendicular to *k*, while using a FDO will result in a cylindrical cut with a central axis parallel to *k*. The SCOT and FDO demonstrate a strong mathematical relation, as both methods rely on the same deformity axis, however, resulting in different cutting planes. These characteristics enable a complementary use when defining the optimal type of osteotomy. This understanding enables a more versatile planning approach when considering factors as the surgical approach, biomechanical characteristics of fixation or soft tissue conditions. The newly introduced stepped FDO facilitates an exact reduction of the bone fragments and potentially expands the clinical applicability of the FDO.

## Introduction

Bone deformities may occur congenital or posttraumatic as a result of malunion of the bone segments and lead to imbalanced joint loading^1,2^. This may further lead to osteoarthritis with severe pain and loss of function, or aesthetic problems^3–5^. Large deformities can lead to quasi-impingement of bones or arise additional tension in ligaments and impair range of motion^2^. This can lead to restrictions in performing daily activities such as drinking, eating, personal hygiene, walking, and other job-related tasks^2^. To treat such bone deformities, corrective osteotomy is an established method^6–11^. Due to the mostly multiplanar malunions with combined torsional and angular deformities, the exact preoperative planning is essential for ideal outcome^8^. After many years of using 2-dimensional imaging as a standard source for planning, it was shown that the use of 3-dimensional bone models extracted from computed tomography (CT) is favorable^12,13^.

Various techniques for planning and executing a corrective osteotomy have been discussed in literature, whereas the closing- and open-wedge osteotomy as well as the single-cut osteotomy (SCOT) the most mentioned ones are^14–18^. The SCOT was introduced in 1952^19^ and improved several times in the following decades, including the description of fundamental mathematical models^20^ and broad presentations of the field of application^14^. The focal dome osteotomy (FDO) is notedly less popular^21^, even though it was already introduced in 1976 by Maquet as the “barrel-vault osteotomy”^22^. This might be due to the more challenging execution of performing a true circular shaped osteotomy and the difficult reduction of the two bone segments in the exact, preoperatively calculated reduction angle. Therefore, the FDO was not broadly studied and discussed in literature especially not in three-dimensional analysis studies. Moreover the close mathematical relationship between the SCOT and FDO has not been described yet.

In the presented paper we would like (1) to describe the close mathematical relationship between the SCOT and FDO and (2) to analyze and introduce a novel technique—the stepped FDO—as a modification of the classic FDO.

## Material and methods

### 3D deformity analysis

The base of any osteotomy planning is the understanding of the bone deformity itself^20^. Such computer-assisted 3D deformity analysis have been described previously in literature repeatedly^2,9,16,17,23,24^. Therefore, we will only summarize the most relevant steps to describe the close trigonometric relationship between the SCOT and FDO.

First, 3D bone models were extracted from the CT data (Siemens SOMATOM Definition AS, Siemens Healthcare, Erlangen, Germany) using a commercial segmentation software (Mimics 19.0; Materialise NV, Leuven, Belgium)^25–27^. The 3D models were then imported into a planning software (CASPA; Balgrist CARD, Zurich, Switzerland) including the reconstruction template. As such reconstruction template different approaches have been described in the literature: the use of the mirrored contralateral anatomy^15,16,28^, the use of a statistical shape model (SSM)^29^, free-hand visual alignment^12^ or any 3D model based on a deformity correction calculation^30^.

**Figure 1:**
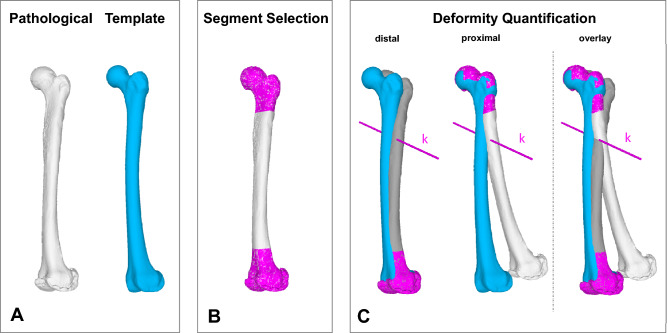
3D deformity analysis. (**A**) A 3D bone model of the pathological bone is extracted from CT data (white) and paired with a reconstruction template (blue, here is a SSM used). (**B**) A proximal and distal segment (magenta) are selected. (**C**) Both segments are superimposed with the reconstruction template. The 3D rotational translational difference between these 2 segments quantify the deformity and is stored in the transformation matrix *T*. The deformity axis *k* is then calculated from the matrix *T*.

To analyze the deformity, a proximal and a distal segment of the bone is matched with the reconstruction template using an iterative closest point algorithm^15,16,31,32^ (see Fig. 1). The 3D rotational and translational difference between these two segments allow the quantification of the deformity and is stored in a 4 × 4 transformation matrix *T* (see Eq. 1)^33^. The three-dimensional deformity axis (represented by the vector *k*) can then be determined from the matrix *T* with the use of a screw displacement axis technique^34–36^. This deformity axis *k* describes a unique axis which allows to perform the complete required transformation as rotation around and translation along its axis^15^. This axis *k* is further used for the planning of the SCOT and FDO.

Transformation Matrix with rotational (R = r_11_–r_33_) and translational (T = T_x_–T_z_) part.

1 $$T = \left[ {\begin{array}{*{20}c} {\begin{array}{*{20}c} {r_{11} } & {r_{12} } \\ {r_{21} } & {r_{22} } \\ \end{array} } & {\begin{array}{*{20}c} {r_{13} } & {T_{x} } \\ {r_{23} } & {T_{y} } \\ \end{array} } \\ {\begin{array}{*{20}c} {r_{31} } & {r_{32} } \\ 0 & 0 \\ \end{array} } & {\begin{array}{*{20}c} {r_{33} } & {T_{z} } \\ 0 & 1 \\ \end{array} } \\ \end{array} } \right]$$

### Single-cut osteotomy

The basic principle of the SCOT is to restore malrotations in all three planes simultaneously. As Sangeorzan et al.^20^ showed, the cutting plane of the SCOT is defined perpendicular to the deformity axis *k*. The transformation is achieved by rotating the distal bone segment over an angle *α* about the deformity axis *k*^1,20,37^ on the cutting plane. The calculation of these parameters is shown in Eq. (2). The SCOT qualifies particularly for deformities with a greater torsion deformity then angular deformity^20^. This configuration leads to an osteotomy plane which approaches the transverse plane (perpendicular to the bone axis). This makes the bone cut small, the post reductional fixation easy and the surgical exposure can be minimal. On the other hand, if the angular deformity is greater than the torsion deformity the osteotomy plane approaches the bone axis itself. Therefore, the surface of the bone cut is getting larger, the post reductional fixation much more difficult and the surgical approach is unpractical. According to Fürnstahl et al.^2^, the SCOT plane is clinically only reasonable, when the translation perpendicular to the cutting plane is negligible (i.e. below 1 mm) and the tangential translation is small. If not, the bone contact surface will be too small or, worse, a gap between the bone parts could be created.

Calculation of the deformity axis (represented by the direction vector k) and the angle α according the rotational part (R = r_11_–r_33_) of the transformation Matrix T (see Eq. 1)

2 $$k = \frac{1}{2\sin \beta }\left| {\begin{array}{*{20}c} {r_{23} - r_{32} } \\ {r_{31} - r_{13} } \\ {r_{12} - r_{21} } \\ \end{array} } \right|, {\upalpha } = \arccos \left( {\frac{1}{2}\left( {r_{11} + r_{22} + r_{33} - 1} \right)} \right)with R = \left( {\begin{array}{*{20}c} {r_{11} } & {r_{12} } & {r_{13} } \\ {r_{21} } & {r_{22} } & {r_{23} } \\ {r_{31} } & {r_{32} } & {r_{33} } \\ \end{array} } \right)$$

### Focal dome osteotomy

**Figure 2:**
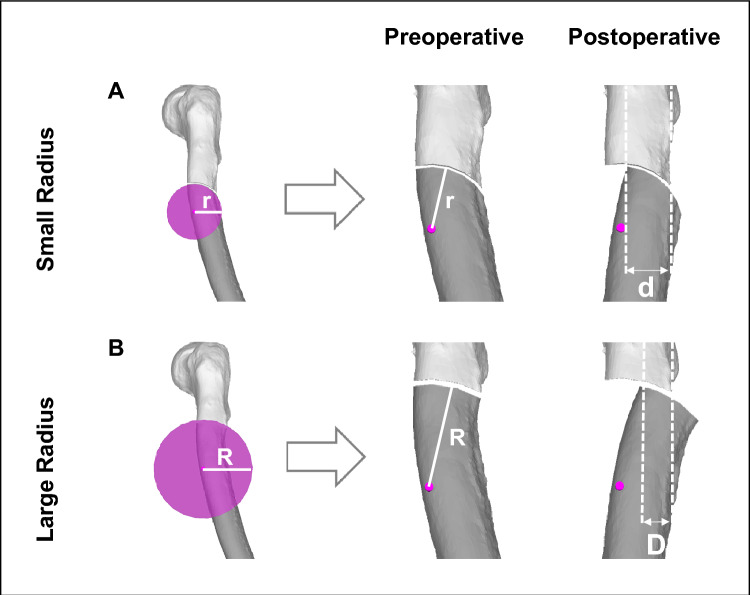
Choice of optimal radius r. The optimal radius *r* of the focal dome osteotomy is chosen as half of the smallest bone diameter, measured through the center axis in any plane perpendicular to the center axis. (**A**) showes an Example with a smaller Radius compared to (**B**). A bigger radius (*R* versus *r*, shown in (**B**)) leads to less bone contact (*d* versus *D*) and more offset.

The basic principle of the FDO is very similar to the SCOT. It restores as well malrotations in all three planes simultaneously. But the transformation is achieved by rotating the distal bone segment around a center axis of a circular (cylindrical) shaped bone cut^14,38,39^ instead of a plane bone cut. The center axis is defined to be parallel to the deformity axis *k.* Therefore as well the rotational axis for SCOT (perpendicular to axis *k*) as well as the rotational axis for FDO (parallel to axis *k*) are closely related to axis *k*. The transformation of FDO is achieved by rotating the distal bone segment over the same angle *α* about the axis *k* (for calculations see Eqs. 1 and 2). Additionally, choosing the optimal radius *r* of the cylindrical bone cut is critical to the biomechanical outcome of the FDO^22,38^. In our opinion, the optimal radius r is chosen as half of the smallest bone diameter, measured through the center axis in any plane perpendicular to the center axis (see Fig. 2). This choice of the radius leads to the greatest possible bone contact area and the smallest axis offset. Many times, the radius is adapted to the anatomical situation and might be slightly bigger.

The FDO qualifies particularly for deformities with a greater angular deformity then torsion deformity^20^. This configuration leads—similar to the SCOT—to an osteotomy cut which approaches the transverse plane (perpendicular to the bone axis). This makes the bone cut as well small, the post reductional fixation easy and the surgical exposure can be minimal. On the other hand, if the torsional deformity is greater than the angular deformity the osteotomy plane approaches the bone axis itself. Therefore, the surface of the bone cut is getting larger, the post reductional fixation much more difficult and the surgical approach is unpractical. According to Paley et al^38^, the deformity regarding axial translation shall be negligible. This is indebted to the single degree of freedom of the FDO restricted to the rotational plane perpendicular to the central axis. If this aspect is neglected, the bone surface will not be congruent or result in a gap formation between the segments. Theoretically, a shift along the center axis of the bone cut is as well possible (perpendicular to the rotating plane), but is virtually always neglected in orthopedic surgery.

**Figure 3:**
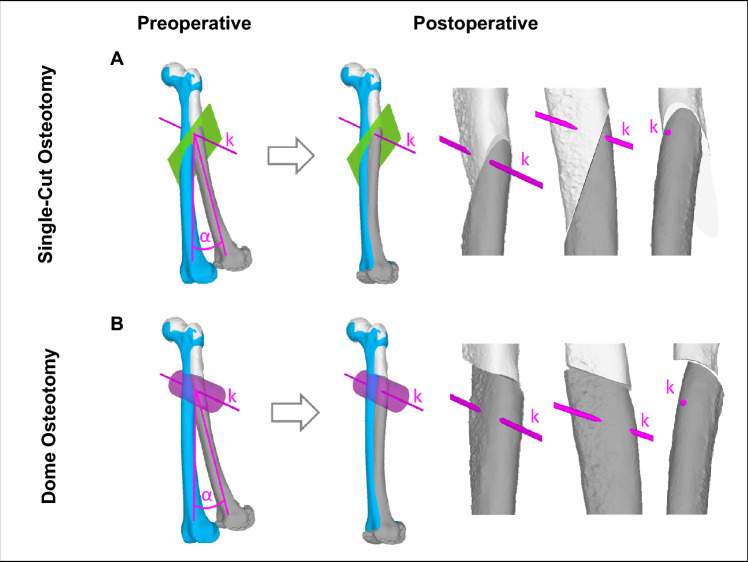
Showing an example of preoperative planning at same deformity for SCOT (**A**) and FDO (**B**). The cutting plane for the SCOT is oriented normal to vector *k* and for FDO it is oriented parallel to vector *k*. Reduction is achieved in both cases by rotating the distal fragment around the deformity axis *k* by the angle *α*.

The similarities between SCOT and FDO are illustrated in Fig. 3. The planning basis for both osteotomies is the deformity axis *k.* The rotational axis for SCOT is perpendicular to axis *k*, whereas the rotational axis for FDO is parallel to axis *k*. For both osteotomies is the reduction done by rotating the distal bone segment over the same angle *α* around the axis *k*. The postoperative situation in Fig. 3 show clearly the different bone cut according to its cutting planes respectively. Another example of the similarities between SCOT and FDO is illustrated in case 1 in section results.

### Stepped focal dome osteotomy

**Figure 4:**
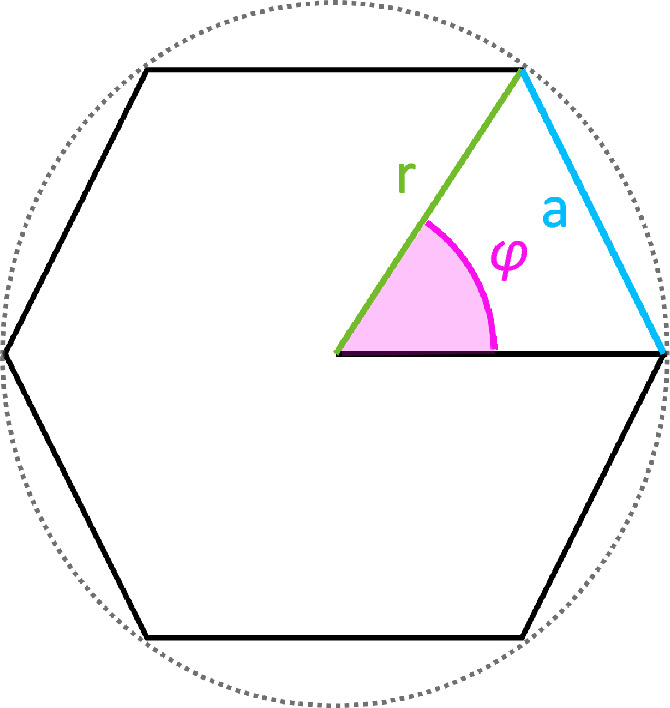
Regular convex polygon (*n* = 6) with radius *r*, center angle *φ* and side *a*.

**Figure 5:**
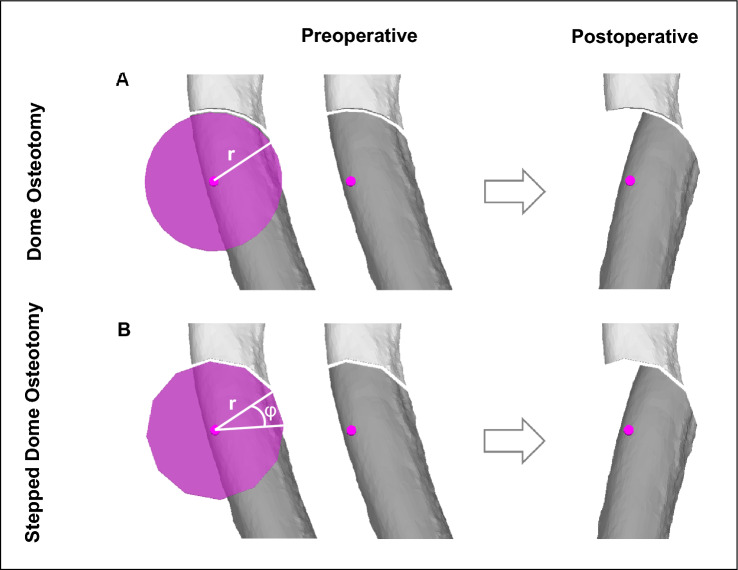
Advantage of the stepped FDO: (**A**) shows a regular FDO. (**B**) shows the same case with a stepped FDO as a regular convex polygon (*n* = 12) with radius *r* and center angle *φ*.

Two of the main challenges when performing a FDO are (1) to cut the dome in a true circular shape and (2) to reduce the two bone segments in the exact, preoperatively calculated angle *α*. To overcome both challenges, we introduce the use of a stepped focal dome osteotomy (stepped FDO). This technical variation simply cuts the dome in the shape of a regular convex polygon instead of a circular shape. A regular convex polygon is a polygon that is equilateral (all sides have the same length) and equiangular (all angles are equal in measure)^40^. The amount of sides/edges (represented as number *n*) correlates with the amount of vertexes and defines the center angle (represented as *φ*)^41^. Figure 4 visualizes these characteristics. The mathematical relation between *n* and *φ* is shown in Eq. (3). If the center angle *φ* of the polygon is chosen as a multiple (*m*) of the needed rotation angle *α*, the stepped focal dome can be rotated stepwise by exact *m*-times to reach the calculated reduction. Furthermore, if the bone deformity and anatomy permit, the radius *r* of the stepped focal dome osteotomy can be adjusted so that the length *a* of the sides of the chosen polygon meet the desired requirements (i.e. width of the saw blade or chisel). As well the arrangement and numbers of the cuts in the bone can be chosen by rotating the polygon around its axis. The planning of the center axis *k* stays unchanged to FDO. Figure 5 shows the advantageous application of the stepped FDO over regular FDO.

Calculation of the center angle φ in a regular convex polygon (n = amount of sides)

3 $$\varphi = \frac{1}{n} \times 360^\circ = \frac{2\pi }{n}$$

### Ethics approval

Written informed consent was obtained from all patients and ethics protocol was approved by the Cantonal Ethics Committee Zurich (BASEC-Nr. 2023–00,389). Therefore the published study complies with the Helsinki Declaration of the World Medical Association.

## Results

The close relationship between SCOT and FDO are summarized in Table 1.

**Table 1:** Summary of the close relationship between SCOT and FDO.

	Single-cut osteotomy (SCOT)	Focal dome osteotomy (FDO)
		
**Deformity Axis and Cutting Plane Orientation**		
Deformity Axis (k)	Deformity axis *k* is determined from the transformation matrix *T* and is the same as for FDO	Deformity axis *k* is determined from the transformation matrix *T* and is the same as for SCOT
Cutting Plane	Cutting Plane: perpendicular to the deformity axis *k*	Cylindrical Cut: parallel to the deformity axis *k*
**Technical Considerations**		
Limitations concerning the Deformity	● Mostly combined torsional (*τ*) and angular (*α*) deformity needed	● Mostly combined torsional (*τ*) and angular (*α*) deformity needed
	● *) Preferably for torsional deformity *τ* greater than angular deformity *α*	● *) Preferably for angular deformity *α* greater than torsional deformity *τ*
	● Negligible translation deformity along the deformity axis *k* (perpendicular to the cutting plane)	● Negligible translation deformity perpendicular to the deformity axis *k* (parallel to the center line)
	● Small translation deformity perpendicular to the deformity axis *k* (tangential to the cutting plane)	
	*) SCOT and FDO are complementary forms of osteotomy and therefore these statements are not absolute limitations for either of the osteotomies	
Surgical Approach	Preferably perpendicular to deformity axis *k*, in-plane with the cutting plane	Preferably parallel to deformity axis *k*, parallel to the cylindrical cut
Bone-Cutting Direction	Perpendicular to deformity axis *k*	Parallel to deformity axis *k*
Post Reductional Fixation	Plate: preferably perpendicular to cutting plane	Plate: preferably perpendicular to deformity axis *k*
	Lag screw: preferably parallel to deformity axis *k*	Nailing: possible fixation
	Nailing: possible fixation	
**Additional Technical Knowledge**		
		Radius *r* of FDO An optimal radius *r* of the osteotomy can be chosen as half of the smallest bone diameter, measured through the center axis in any plane perpendicular to the center axis (see Fig. 2)

**Figure 6:**
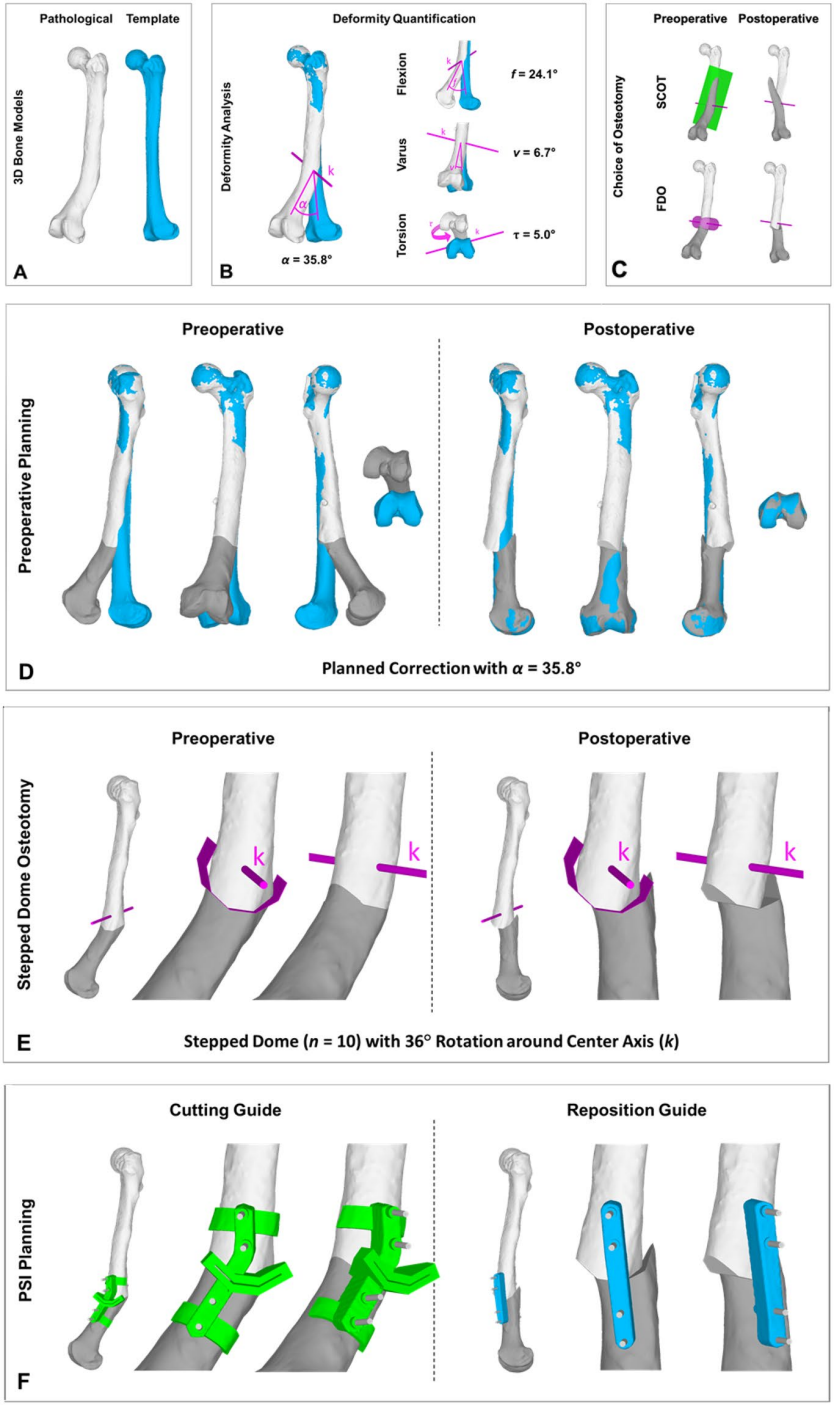
Illustration of a correction osteotomy by a stepped FDO for a posttraumatic deformity of a left distal femur. (**A**) 3D bone model of the pathological bone (white) and the template (contra-lateral side, blue). (**B**) Deformity quantification while overlaying the pathological bone (white) and the template (blue). (**C**) Choice of the osteotomy between SCOT and FDO. (**D**) Preoperative planning with *α* = 35.8° shows the preoperative and postoperative situation with the pathological bone (proximal: white, distal: grey) and template (blue). (**E**) Preoperative planning with stepped FDO (*n* = 10, *α* = 36°) around center axis *k*. (**F**) PSI planning for cutting and reduction guides.

**Figure 7:**
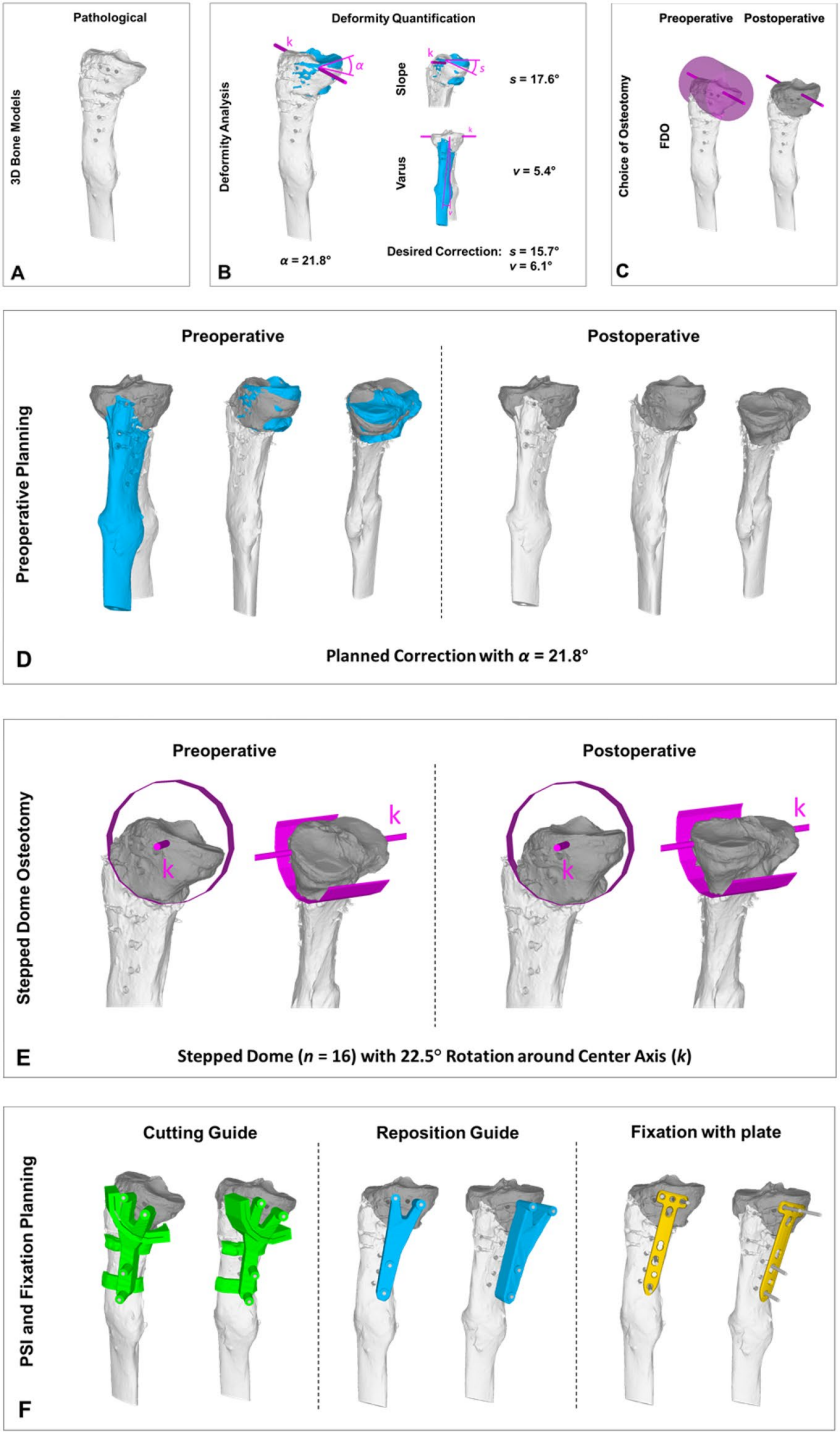
Illustration of a correction osteotomy by a stepped FDO for a posttraumatic and postinterventional deformity of a right proximal tibia. (**A**) 3D bone model of the pathological bone (white). (**B**) Deformity quantification while overlaying the pathological bone (white) and the template (blue). (**C**) Choice of the osteotomy as FDO. (**D**) Preoperative planning with *α* = 21.8° shows the preoperative and postoperative situation with the pathological bone (proximal: grey, distal: white) and template (blue). (**E**) Preoperative planning with stepped FDO (*n* = 16, *α* = 22.5°) around center axis *k*. (**F**) PSI planning for cutting and reduction guides and planned plate position.

Figures 6 and 7 show two applications of the stepped FDO to illustrate the technical feasibility and its advantages. In case 1 (Fig. 6) a posttraumatic deformity of a left distal femur has been realigned to its contra-lateral side. The deformity analysis showed a deviation in flexion of 24.1°, in varus of 6.7° and in torsion of 5.0°. The total deformity angle *α* yields 35.8°. Due to the large flexion deformity, the better postoperative bone congruency, the smaller surgical approach and the easier post reductional fixation, we chose to plan a FDO over a SCOT. The needed correction angle of almost 36° allowed to plan a stepped FDO with a polygon of 10 sides (*n* = 10). Therefore the center angle *φ* was equal to 36°. For the reduction, the distal bone fragment had to be rotated 1 step (*m* = 1). The cutting and reduction were assisted with patent-specific instrumentation (PSI). In case 2 (Fig. 7) a posttraumatic deformity of a right proximal tibia was initially corrected with an opening-wedge high tibial osteotomy (HTO), but due to delayed union the correction was faulted. The deformity analysis showed a posterior slope of 17.6° and a varus of 5.4°. The desired correction was decrease of slope by 15.7° and valgisation by 6.1°. The total deformity angle *α* was 21.8°. Due to the primarily slope reduction, the easier surgical approach, the history of the patient and the easier post reductional fixation, we chose to plan a FDO. The needed correction angle of almost 22° allowed to plan a stepped FDO with a polygon of 16 sides (*n* = 16). Therefore the center angle *φ* was equal to 22.5°. For the reduction, the distal bone fragment had to be rotated 1 step (*m* = 1). The cutting and reduction were assisted with patent-specific instrumentation (PSI). The plate for the post reductional fixation was planned as well properatively.

## Discussion

We illustrated in this paper the close relationship between SCOT and FDO as both methods rely on the same deformity axis *k*. The contrary direction of the according cutting planes (SCOT: cutting plane perpendicular to axis *k*, FDO: cylindrical cut parallel to axis *k*) leads to as well contrary indications for each osteotomy type (SCOT: particularly for deformities with a greater torsion deformity then angular deformity, FDO: rather for deformities with a greater angular deformity then torsion deformity). Both these configurations lead then to an osteotomy plane approaching the transverse/axial plane (perpendicular to the bone axis). This leads to small bone cut, easy post reductional fixation and minimal surgical exposure. On the other hand, if these recommendations are violated (e.g. SCOT for deformities with a greater angular deformity then torsion deformity) the osteotomy plane approaches the bone axis itself. Therefore, the surface of the bone cut is getting larger, the post reductional fixation much more difficult and the surgical approach is unpractical. These contrary indications for SCOT and FDO, lead as well to contrary technical considerations (surgical approach, bone cutting direction, post reductional fixation—see Table 1). All these contrary characteristics make the SCOT and FDO to complementary tools in the planning process while choosing the optimal type of osteotomy. Even though both types of the osteotomies are well known since a long time, their mathematical and clinical relations have never been descripted in literature in this extent.

The execution of the SCOT and FDO is sometimes quite challenging due to the multidirectional rotation of the deformity axis *k* in space. The guidance with patient-specific instrumentation (PSI) can be an essential help for the correct orientation of the cutting plane by SCOT^8,12,16,17,25^. For the best guidance of the circular cutting plane of the FDO exists no consensus in the literature. Our proposal is either an PSI with multiple drill-holes in a dome shaped arrangement or to set a K-wire with a PSI in the exact direction of the pre-calculated rotation axis *k* and use a circular saw with the corresponding radius *r*. But it still remains often an imprecise step of the operation. We as well propose to use a reduction guide to perform the needed circular reduction accurately enough (see Figs. 6 and 7).

The newly introduced stepped FDO can eliminate the two biggest challenges of performing a FDO by cutting the dome in a regular convex polygon rather than in a true circular dome shaped matter. The internal angle of the polygon is then chosen as the multiple *m* of the needed rotation angle *α* around the center axis. This leads to an easy reduction while rotating the distal bone fragment exact *m*-times, like *m* steps in a rack-wheel. Additionally the length, numbers and arrangement of the sides of the polygon can be adjusted to the local anatomy by choosing an appropriate radius and by rotating the polygon around its axis. These properties make the stepped FDO a suitable and valuable additional type of osteotomy.

In conclusion, we could illustrated in this paper for the first time in literature (1) the strong relation between SCOT and FDO since both methods relay on the same axis computed from the deformity analysis. These characteristics make them to complementary tools while choosing the optimal type of osteotomy. (2) The newly introduced stepped FDO is technically and clinically feasible and can eliminate the biggest challenges of performing a FDO. Therefore it makes the FDO to a more valuable osteotomy option.

## Data Availability

The datasets used and analyzed during the current study are available from the corresponding author on reasonable request.
